# Supplementary material for: The Longitudinal Analysis of Convergent Antibody VDJ Regions in SARS-CoV-2-Positive Patients Using RNA-Seq
Source: Viruses. 2023 May 26;15(6):1253. doi: 10.3390/v15061253 (PMC10304081; doi:10.3390/v15061253)
Supplement: Supplementary file 1 [file viruses-15-01253-s001.zip › zip supplemental/Supplementary_Fig1_Fig2_Table_Legend[1].pdf]

Supplementary Table Legends and Figures

Supplementary Table S1: Excel spreadsheet lists compilation of all 629,133 reconstructed immunoglobulin heavy variable domain sequences used in this study. Column (A) indicates the *IGHV* gene, (B) the CDR3 sequence, and (C) *IGHJ* gene respectively. Column (D) indicates which patient group the sequence is from. Column (E) indicated whether that VDJ sequence occurred more than once in that patient group (commonVDJ), or only in one individual patient (singleVDJ) of the group. Column (F) indicates our ID number for the shared VDJ. Column (G) indicates the total number of patient groups the VDJ sequence was detected in. Column (H) indicated whether that shared VDJ is a commonVDJ to all groups it is found in, only occurred as a singleVDJ in all groups it was present in, or as combination of commonVDJ and singleVDJ (mixed). Column (I) indicated the number of patients the VDJ was found in per group (always 1 if a singleVDJ).

Supplementary Table S2: The table shows the 17 IGC-VDJs that match with antibodies from the CoV-AbDab database. Column A lists the serial IGC-VDJ number of the matching antibody. Column (B) indicates the IGHV gene, (C) the CDR3 sequence, and (D) the IGHJ gene respectively. Column (E) shows which SARS-CoV-2 variant the antibody binds to (all the IGC-VDJs are found in antibodies that bind SARS-CoV-2 wildtypes) while Column (F)shows which variants the antibody does not bind to. Column G indicates the variants in which the matching antibody neutralizes to, and Column (H) indicates which variants the antibody does not neutralize to. Column I list the references for each of the antibodies.

Supplementary Table S3: The table lists the common antibodies' *IGHV*, CDR3, and *IGHJ* gene sequence as well as indicating which group each common antibody is found in. Column A lists the number of patients that have the specific common CDR3 sequence listed in column C. Column B indicates the *IGHV* usage for immunoglobulins in SARS-CoV-2 patients. Column D indicates the *IGHJ* usage for each patient. Column E lists the group number that each patient is from.

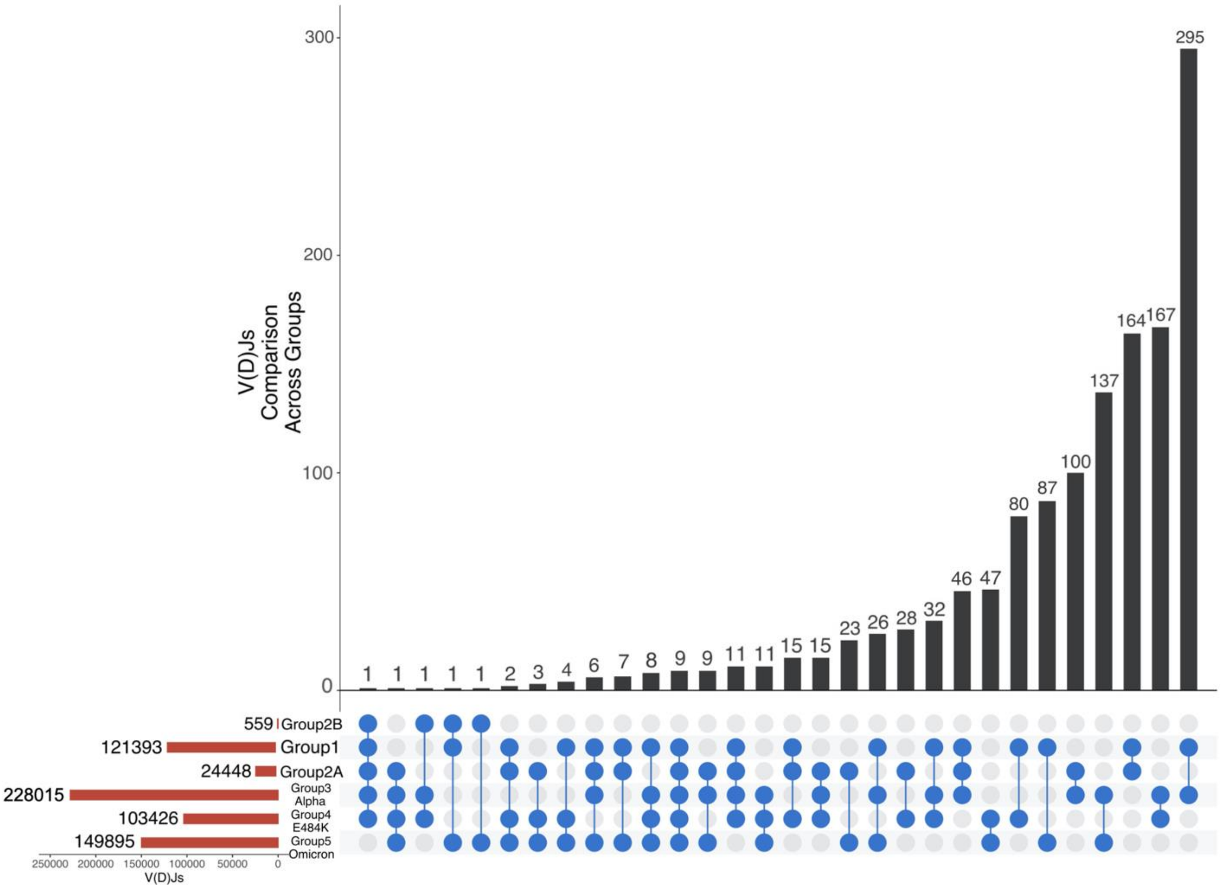

Supplementary Figure 1: A longitudinal comparison between shared VDJs (more than one patient sharing the same V gene, J gene, and CDR3 sequence across all groups) across patients from all five groups. The red bars at the bottom left show the total number of VDJs for each group. The connected blue dots indicate the shared VDJs across the groups that are indicated with a blue dot. The black bars at the top show the number of shared VDJs across the groups that were shown by the connected blue dots.

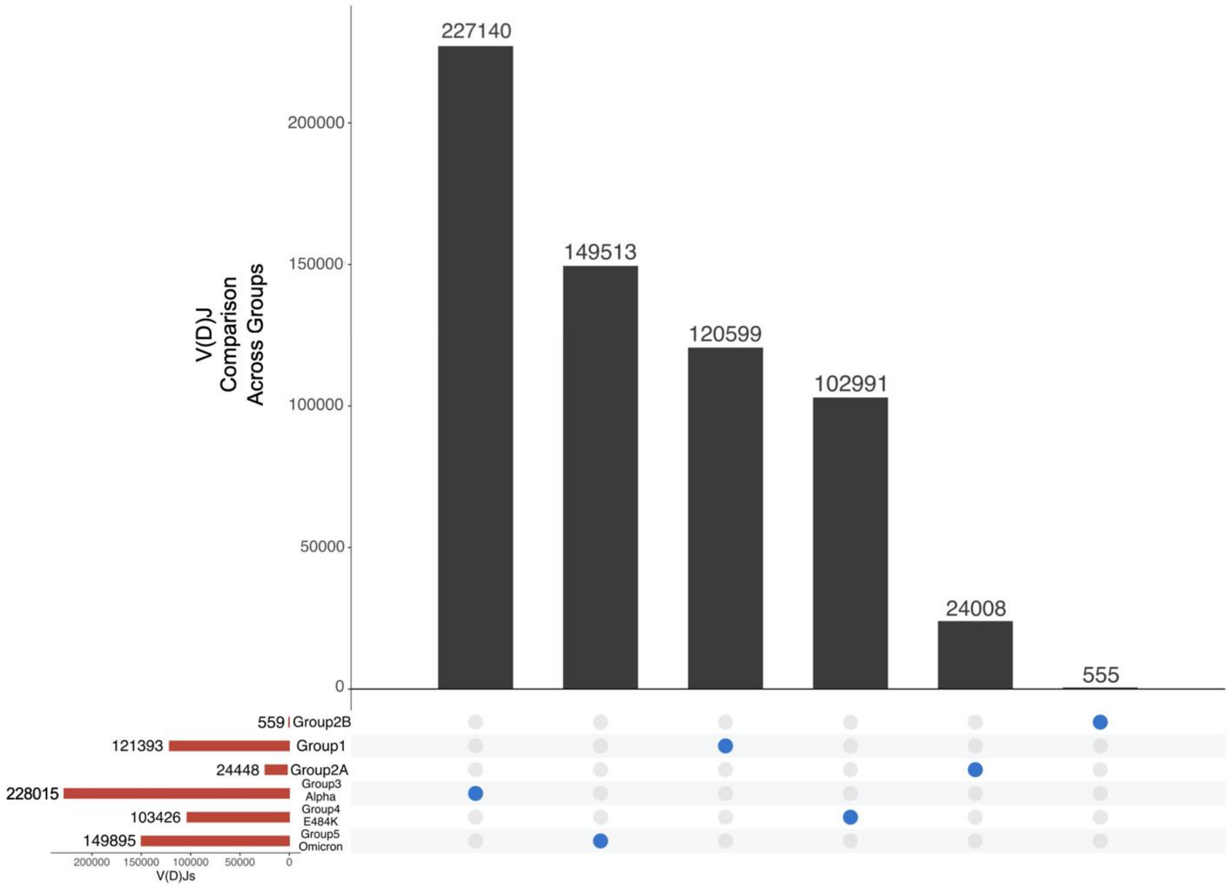

Supplementary Figure 2: VDJ sequences that appear in only one patient . The red bars at the bottom left indicate the total number of unique VDJ sequences reconstructed for each patient group for the entire study. The black bars at the top indicate the number of those VDJs sequences that was id single in the group indicated by the blue dots.
